# Impact of age on thromboembolic events in patients with non‐valvular atrial fibrillation

**DOI:** 10.1002/clc.23293

**Published:** 2019-11-15

**Authors:** Yun Gi Kim, Jong‐Il Choi, Ki Yung Boo, Do Young Kim, Yeji Hong, Min Sun Kim, Kwang‐No Lee, Jaemin Shim, Jin Seok Kim, Young‐Hoon Kim

**Affiliations:** ^1^ Division of Cardiology, Department of Internal Medicine Korea University Medicine Anam Hospital Seoul Republic of Korea; ^2^ Division of Medical Statistics Korea University College of Medicine Seoul Republic of Korea

**Keywords:** age, atrial fibrillation, ischemic stroke, thromboembolic complication, transient ischemic attack

## Abstract

**Background:**

Age is a well‐established risk factor for thromboembolic events in patients with atrial fibrillation (AF). However, the mechanism underlying the association between age and thromboembolic events in AF remains unknown.

**Methods:**

The prognostic value of age as a risk factor for thromboembolic events was analyzed using data from the Korean National Health Insurance Service (NHIS). In a large‐scale single‐center registry, cardiac hemodynamic parameters were examined to elucidate the cause of increased risk of thromboembolic events in older patients.

**Results:**

NHIS sample cohort data including 5896 patients with AF revealed that the risk of thromboembolic complication differed significantly according to age despite equal CHA_2_DS_2_‐VASc score. In the registry of 2801 patients, age showed significant correlations with left atrium (LA) diameter, LA volume, E/e′, pulmonary artery pressure, and LA appendage flow velocity. Older patients had a significantly higher prevalence of spontaneous echocontrast (odds ratio [OR] = 1.030; *P* < .001). Age (OR = 1.031; *P* < .001), E/e′ (OR = 1.065; *P* = .004), and LA appendage flow velocity (OR = .988; *P* = .009) were significant predictors for thromboembolic events in multivariate analyses. In data from the NHIS, CHA_2_DS_2_‐VASc score did not outperform age to predict thromboembolic events.

**Conclusions:**

Age is a significant risk factor for thromboembolic events in patients with AF, and old age is associated with adverse cardiac hemodynamics. This study suggests that older patients with AF are at high risk of thromboembolic events regardless of CHA_2_DS_2_‐VASc score.

**Abbreviations**
AAD: antiarrhythmic drug
AF: atrial fibrillation
AUC: area under curve
BMI: body mass index
EF: ejection fraction
HR: hazard ratio
LA: left atrium
LAA: left atrial appendage
LR: late recurrence
LV: left ventricle
OR: odds ratio
RFCA: radiofrequency catheter ablation
ROC: receiver operating characteristic
SEC: spontaneous echocontrast
TEE: transesophageal echocardiography
TIA: transient ischemic attack.
TTE: transthoracic echocardiography

## 1 INTRODUCTION

A substantial proportion of ischemic stroke, transient ischemic attack (TIA), and systemic embolism are caused by atrial fibrillation (AF).^1^, ^2^, ^3^ The thromboembolic events in AF patients are associated with significantly increased morbidity and mortality. Significant efforts are made to identify high risk patients and prevent these catastrophic complications.^4^, ^5^, ^6^, ^7^, ^8^, ^9^ The CHA_2_DS_2_‐VASc scoring system is the most common system used to stratify risk of thromboembolic events.^6^, ^7^, ^10^, ^11^ CHA_2_DS_2_‐VASc score is calculated by summing seven components: congestive heart failure, hypertension, age, diabetes, previous stroke/TIA/systemic embolism, vascular disease, and sex category.^7^, ^12^ For age, one point is given for age 65‐74 and two points are given for patients equal or older than 75 years.^7^ The use of anticoagulation therapy to prevent thromboembolic events in patients with AF is guided by the CHA_2_DS_2_‐VASc scoring system, and anticoagulation should be recommended to patients with CHA_2_DS_2_‐VASc score ≥ 2.^10^, ^11^ The CHA_2_DS_2_‐VASc system can identify patients at low risk for thromboembolic events.^13^ However, CHA_2_DS_2_‐VASc is not perfect and the C‐statistic was 0.606 according to the Euro Heart Survey on AF.^7^, ^14^ A previous study by Chao et al. showed that assigning additional points to those older than 50 years improved the C‐statistic and enabled identification of low risk patients more precisely in East Asian patients.^15^ A recent study also demonstrated that age is the most powerful predictor of ischemic stroke in patients with AF.^16^ However, the mechanism underlying age as a dominant risk factor for thromboembolic events in patients with AF is not fully understood.

We aimed to evaluate the relative importance of age as a risk factor for thromboembolic events and to elucidate the underlying pathophysiology of the association between age and thromboembolic events. We used both the Korean National Health Insurance Service (K‐NHIS) sample cohort data and the Korea University Medical Center Anam Hospital radiofrequency catheter ablation (RFCA) registry (KUMC registry).

## 2 METHODS

### 2.1 A nationwide sample cohort

The NHIS is the single medical insurer in the Republic of Korea managed by the government. The majority of Korean people (97.1%) are mandatory subscribers, and the database is open to medical researchers. The K‐NHIS sample cohort was created and released by NHIS in 2014 and contains 1 025 340 individuals representing the entire Korean population from the beginning of 2002, accounting for 2.2% of the entire Korean population in the K‐NHIS system. Patients included in the K‐NHIS sample cohort were followed until 2013, and the database contains demographics, diagnosis codes, use of inpatient and outpatient services, pharmacy dispensing claims, and mortality data. The diagnosis of AF required one inpatient or two outpatient records of international classification of disease, tenth revision (ICD‐10) codes in the database. Years 2002 to 2004 were used as a screening period, and patients newly diagnosed with AF beginning from January 2005 were included in the analysis. Diagnosis of hypertension, diabetes, heart failure, vascular disease, ischemic stroke, TIA, and systemic embolism were performed using data of the screening period. Patients diagnosed with ischemic stroke, TIA, and systemic embolism in the screening period were excluded from the analysis to prevent misclassification of old thromboembolic events as newly diagnosed thromboembolic events. The exact diagnosis codes for AF, hypertension, diabetes, heart failure, vascular disease, ischemic stroke, TIA, and systemic embolism are presented in Table S1. If a patient was prescribed anticoagulants for more than 6 months after the diagnosis of AF, he or she was considered to have had anticoagulation therapy. The study protocols were approved by the official review committee of the Korean government.

### 2.2 Registry‐based data

The KUMC registry consisted of consecutive patients with AF who underwent their first RFCA in KUMC Anam Hospital from June 1998 to December 2017. All patients who underwent RFCA in the institution were included, and there were no specific exclusion criteria. The exact profile of the registry is reported elsewhere.^8^, ^17^ The current study was approved by the institutional review board, which ensured appropriate ethical and bioethical conduct. Informed consent was waived since this was a retrospective study. The protocol was consistent with the ethical guidelines of the 2008 Helsinki Declaration.

In the KUMC registry, both transthoracic echocardiography (TTE) and transesophageal echocardiography (TEE) were performed before RFCA. Left atrial (LA) size, left ventricular (LV) ejection fraction (EF), mitral valve inflow velocity (E), and mitral annular tissue velocity (e′) were measured during TTE evaluation. For TEE evaluation, multiple views (high esophageal 0°, 45°, 60°, and 120° views) were obtained and emptying (forward), filling (backward), and average flow velocity of the LA appendage (LAA) were measured. The presence of spontaneous echocontrast (SEC) or thrombus was carefully evaluated. SEC was divided into grades of very mild (minimal echogenicity, only detectable transiently, or increasing gain setting required for the detection), mild (detectable without increasing gain setting), moderate (dense, swirling echogenic material; echogenic signal is dense in LAA compared to LA), or severe (dense, swirling echogenic material; echogenic signal is equivocal in LAA, and LA). Dense SEC was defined as a composite of moderate and severe SEC.

### 2.3 Study end points

The occurrence of ischemic stroke or TIA was the end point in analysis using the K‐NIHS sample cohort. The relative influences of age and other risk factors were evaluated through various approaches. In analysis with the KUMC registry, previous history of ischemic stroke, TIA, and systemic embolism was the end point. The impact of age and other clinical risk factors on thromboembolic events was evaluated. The influence of various cardiac hemodynamic parameters measured with TTE and TEE was examined, and the relationship between age and cardiac hemodynamics was also analyzed.

### 2.4 Statistical analysis

Continuous variables were described as mean ± SD, and were compared using a Student's *t*‐test. Categorical variables were presented as percentile values, and were compared with a chi‐square test or Fisher's exact test as appropriate. Pearson product‐moment correlation analysis was performed to examine correlation between two continuous variables. Thromboembolic event‐free survival was depicted by Kaplan‐Meier survival curve analysis, and the difference between groups was assessed using a log‐rank test. Cox regression analysis was performed to calculate the hazard ratio (HR) and 95% confidence interval (CI). Receiver operating characteristic (ROC) curve analysis with calculation of the area under the curve (AUC) was performed to evaluate the efficacy of CHA_2_DS_2_‐VASc score and to predict thromboembolic events. Comparison of AUCs of two ROC curves was performed using the statistical method suggested by Hanley and McNeil.^18^ Logistic regression analysis was performed to calculate the odds ratio (OR) with 95% CI. All significance tests were two‐tailed, and *P* values equal to or less than .05 were considered statistically significant. All statistical analyses were performed with SPSS version 21.0 (IBM, Armonk, NY).

## 3 RESULTS

### 3.1 Patients

Among 1 025 340 patients in the K‐NHIS sample cohort, 5896 patients were diagnosed with AF from January 2005 to December 2012. Baseline demographic data including individual components of the CHA_2_DS_2_‐VASc score were obtained during the screening period from January 2002 to December 2004. Baseline characteristics of the study population are summarized in Table S2. Male patients comprised 54.7% of the study population, and mean CHA_2_DS_2_‐VASc score was 2.99 ± 1.96. The KUMC registry included a total of 2801 patients with AF who underwent RFCA for the first time. Mean age and CHA_2_DS_2_‐VASc score was 55.58 ± 10.97 and 1.25 ± 1.26, respectively. Baseline patient demographics are described in Table S2. TTE, TEE, and cardiac MRI was performed in 2742 (97.89%), 2580 (92.10%), and 932 (33.27%) patients, respectively.

### 3.2 K‐NHIS sample cohort

**Figure 1:**
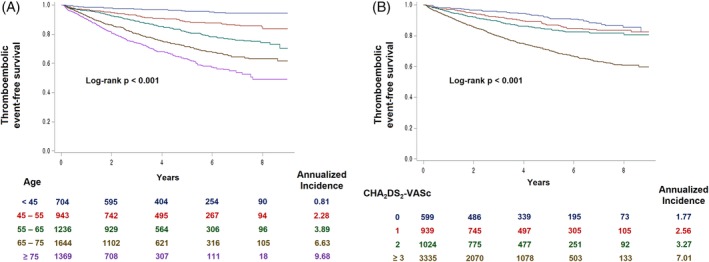
Risk of thromboembolic events stratified by age and CHA_2_DS_2_‐VASc score. Risk of thromboembolic events increased gradually as age, A, and CHA_2_DS_2_‐VASc score, B, increased. CI, confidence interval; HR, hazard ratio

**Figure 2:**
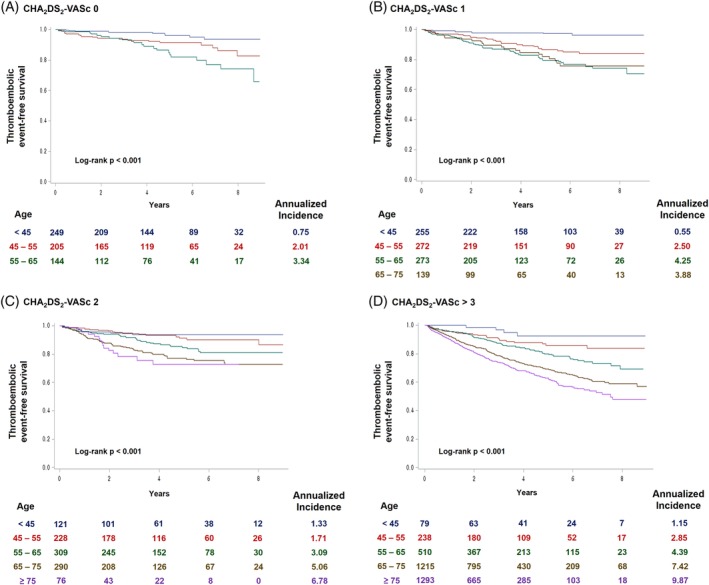
Impact of age within equal CHA_2_DS_2_‐VASc score. Risk of thromboembolic events was significantly influenced by age in patients with CHA_2_DS_2_‐VASc score 0, A, 1, B, 2, C, and ≥ 3, D. CI, confidence interval; HR, hazard ratio

Cumulative incidence of thromboembolic events differed significantly according to age and CHA_2_DS_2_‐VASc score (Figure 1A,B). Age had a significant impact on thromboembolic events among patients with equal CHA_2_DS_2_‐VASc scores. In patients with CHA_2_DS_2_‐VASc score 0, patients <45 years old had significantly lower risk of thromboembolic events compared to patients 45‐55 or 55‐65 years old (Figure 2A). Patients with CHA_2_DS_2_‐VASc scores of 1, 2, and ≥ 3 also showed significantly different risks of thromboembolic events according to age group (Figure 2B‐D). Patients under 45 years had a low risk of thromboembolic events irrespective of the CHA_2_DS_2_‐VASc score (Figure 2A‐D).

**Figure 3:**
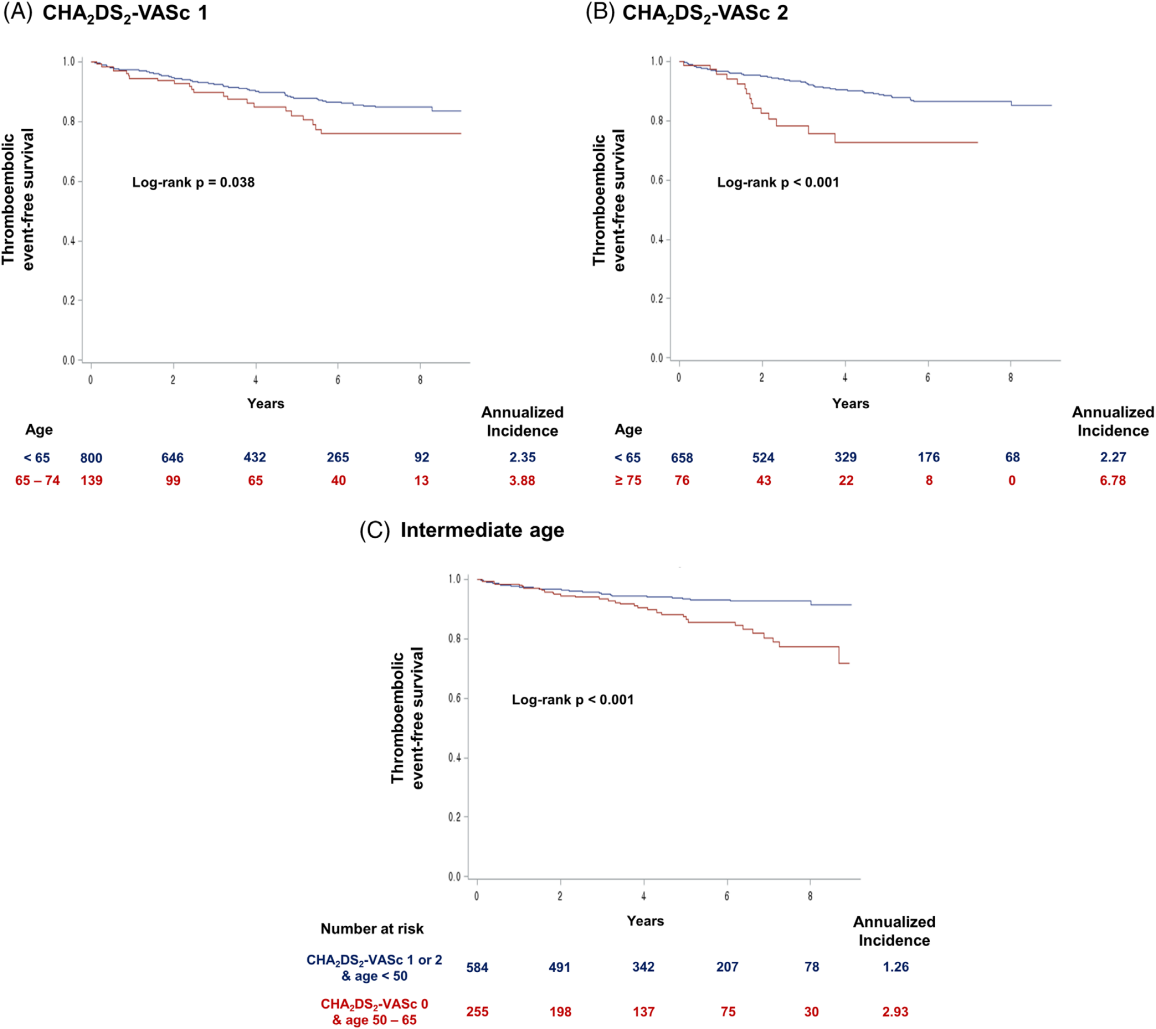
Relative risk of age compared with other risk factors. Increase in CHA_2_DS_2_‐VASc score due to age criteria was associated with greater risk compared to other risk factors in patients with CHA_2_DS_2_‐VASc score 1, A, or 2, B. Age had a significant impact on thromboembolic events even across different CHA_2_DS_2_‐VASc scores, C. CI, confidence interval; HR, hazard ratio

**Figure 4:**
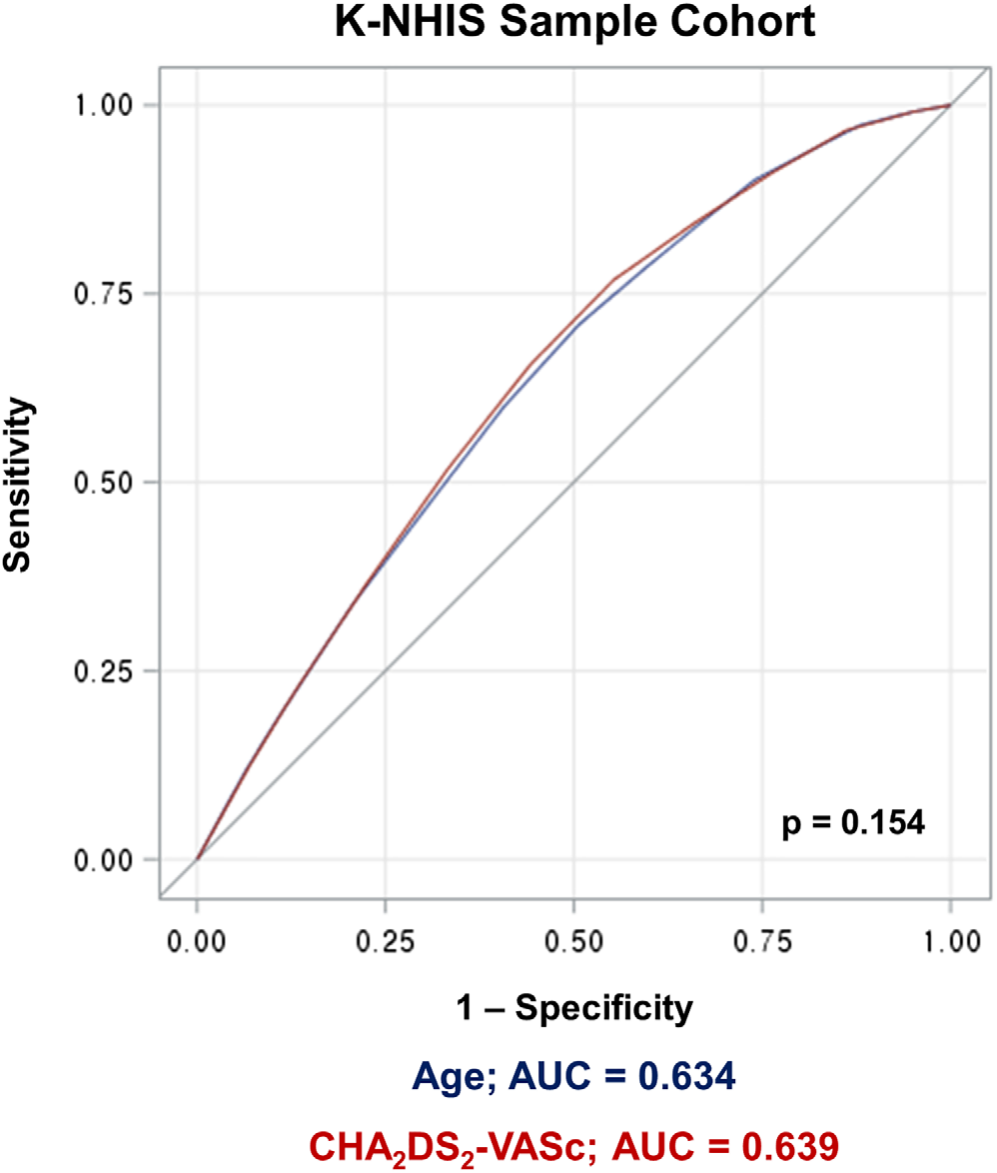
ROC curve analysis: age vs CHA_2_DS_2_‐VASc score. CHA_2_DS_2_‐VASc score and age showed similar efficacy to predict thromboembolic events in K‐NHIS sample cohort data. AUC, area under curve; CI, confidence interval; ROC, receiver operating characteristic

In patients with CHA_2_DS_2_‐VASc score 1, those with 1 point due to age (65 ≤ age < 75) had significantly higher cumulative incidence of thromboembolic events compared to those with CHA_2_DS_2_‐VASc score of 1 for reasons other than age criteria (HR = 1.635; 95% CI = 1.02‐2.61; *P* = .038; Figure 3A). Patients with two points in CHA_2_DS_2_‐VASc score for age older than 75 years had a significantly higher risk of thromboembolic events compared to patients with two points for reasons other than age (HR = 2.713; 95% CI = 1.54‐4.78; *P* < .001; Figure 3B). In subgroup analysis, the difference in the risk of thromboembolic events between the two aforementioned groups was significant in patients who did not take anticoagulants (HR = 3.403; 95% CI = 1.83‐6.33; *P* < .001) but was not significant in patients who took anticoagulants (HR = 1.121; 95% CI = 0.26‐4.82; *P* = .878). Patients with CHA_2_DS_2_‐VASc score of 0 and aged between 50 and 65 years showed significantly higher risk of thromboembolic events compared to patients with CHA_2_DS_2_‐VASc score of 1 or 2 but less than 50 years old (HR = 2.297; 95% CI = 1.43‐3.70; *P* < .001; Figure 3C). Patients between 50 and 65 years had significantly higher risk of thromboembolic events compared to those under 50 years (HR = 2.508, 95% CI = 1.88‐3.34; *P* < .001) in multivariate analysis including individual components of CHA_2_DS_2_‐VASc score. On ROC curve analysis, CHA_2_DS_2_‐VASc score and age showed similar efficacy to predict future thromboembolic events (AUC = 0.639 vs 0.634; *P* = .154; Figure 4).

### 3.3 KUMC registry

In KUMC registry, age (OR = 1.031; 95% CI = 1.014‐1.048; *P* < .001), E/e′ (OR = 1.065; 95% CI = 1.021‐1.110; *P* = .004), and LAA flow velocity (OR = 0.988; 95% CI = 0.980‐0.997; *P* = .009) were independent risk factors associated with thromboembolic events in multivariate analysis (Table 1). In correlation analysis, old age was associated with significantly increased E/e′ (0.362; *P* < .001) and decreased LAA flow velocity (r = −0.159; *P* < .001) (Table 2). Older patients had a significantly higher prevalence of spontaneous echocontrast (OR = 1.030 for every one year increase in age; *P* < .001).

**Table 1:** Clinical and echocardiographic risk factors for thromboembolic events

	Univariate analysis			Multivariate analysis^a^			
	HR	95% CI	*P* value	HR	95% CI		*P* value
Age	1.041	1.027‐1.054	.000	1.031	1.014–1.048		.000
LA diameter	1.033	1.010‐1.055	.004	0.992	0.963‐1.022		.587
LV EF	0.975	0.956‐0.994	.012	0.984	0.956‐1.013		.278
PAP	1.006	0.979‐1.034	.683				
E/e′	1.095	1.058‐1.133	.000	1.065	1.021–1.110		.004
LAA flow velocity	0.987	0.980‐0.994	.000	0.988	0.980–0.997		.009
SEC	1.487	1.085‐2.039	.014	0.966	0.648‐1.439		.863
CHA_2_DS_2_‐VASc	1.226	1.095‐1.373	.000				

Abbreviations: CI, confidence interval; E/e′: E over e′; HR, hazard ratio; LA, left atrium; LAA, LA appendage; LV EF, left ventricular ejection fraction; PAP, pulmonary artery pressure; SEC, spontaneous echocontrast.

a The model included age, LA diameter, LV EF, E/e′, LAA flow velocity, SEC, sex, congestive heart failure, hypertension, diabetes mellitus, and vascular disease.

**Table 2:** Correlation among age and cardiac hemodynamic parameters

	All patients (N = 2801)		Paroxysmal AF (n = 1656)		Non‐paroxysmal AF (n = 1145)	
	r	*P* value	r	*P* value	r	*P* value
LA diameter	0.230	< .001	0.243	< .001	0.211	< .001
LA volume	0.254	< .001	0.334	< .001	0.202	< .001
LV EF	0.020	.296	0.042	.095	0.029	.332
PAP	0.223	< .001	0.255	< .001	0.175	< .001
E/e′	0.362	< .001	0.371	< .001	0.352	< .001
LAA flow velocity	−0.159	< .001	−0.175	< .001	−0.151	< .001

Abbreviations: E/e′, E over e′; LA, left atrium; LAA, LA appendage; LV EF, left ventricular ejection fraction; PAP, pulmonary artery pressure.

## 4 DISCUSSION

The major findings of our study are summarized as follows: (a) risk of thromboembolic events in patients with AF differs significantly according to age; (b) increased CHA_2_DS_2_‐VASc score due to age criteria is associated with higher risk of thromboembolic events; (c) intermediate age group (50‐65) has an increased risk of thromboembolic events although they do not receive age points in the CHA_2_DS_2_‐VASc scoring system; (d) old age is associated with adverse cardiac hemodynamics; (e) old age, increased E/e′, and decreased LAA flow velocity are independent risk factors for thromboembolic events in patients with AF. The current study utilized not only insurance data but also registry data to analyze echocardiographic parameters and clinical factors.

### 4.1 Age and thromboembolic events

Age is a critical risk factor for thromboembolic events in patients with AF.^6^, ^15^, ^16^, ^19^ In accordance with previous studies, our study showed that age is the most important risk factor for thromboembolic events in AF patients with low to intermediate CHA_2_DS_2_‐VASc score (0‐2).^6^, ^20^, ^21^, ^22^ In addition, our study also demonstrated that age is a critical risk factor in patients with AF and high CHA_2_DS_2_‐VASc score (≥ 3).

Anticoagulation is recommended in patients with CHA_2_DS_2_‐VASc score ≥ 2. However, previous report by Chao et al. suggested that not all risk factors in CHA_2_DS_2_‐VASc score carry an equal risk and age criteria (65 to 74 years) was associated with the highest stroke risk.^23^ Intermediate age (50 to 64 years) was also associated with increased risk of ischemic stroke.^21^, ^22^ Our study also found that patients who had a CHA_2_DS_2_‐VASc score of 1 for being older than 65 years showed a greater risk of thromboembolic events than patients with CHA_2_DS_2_‐VASc score of 1 due to other risk factors (Figure 2A). Previous study by Freiberg et al. suggested that routine anticoagulation therapy is not justified in patients with CHA_2_DS_2_‐VASc score of 1.^24^ However, our study indicate that the risk of thromboembolic event differ significantly depending on age in patients with CHA_2_DS_2_‐VASc score of 1 and certain subgroup of patients might benefit from anticoagulation therapy (Figure 2B). Furthermore, we also revealed that age had a significant influence on thromboembolic events beyond CHA_2_DS_2_‐VASc scores. Patients with CHA_2_DS_2_‐VASc score of 0 but between 50 and 65 years old were at significantly higher risk of thromboembolic events compared to patients with CHA_2_DS_2_‐VASc score of 1 or 2 but under 50 years.

We revealed that young patients (< 45) were at low risk of thromboembolic events irrespective of CHA_2_DS_2_‐VASc score and that the risk and benefit profile should be reviewed before giving anticoagulants to these patients. Therefore, anticoagulation therapy based solely on CHA_2_DS_2_‐VASc score might miss a subgroup of patients who require anticoagulation therapy despite low CHA_2_DS_2_‐VASc score and can also result in overtreatment of patients who might not need anticoagulation therapy despite high CHA_2_DS_2_‐VASc score. Patients with a CHA_2_DS_2_‐VASc score of 2 due to age ≥ 75 years had a significantly higher risk of thromboembolic events than patients with a CHA_2_DS_2_‐VASc score of 2 due to factors other than age criteria. Anticoagulation is strongly recommended in patients older than 75 years.

### 4.2 Age and cardiac hemodynamics

The KUMC registry included both TTE and TEE data for a substantial proportion of patients. Analysis of this registry revealed that older patients had significantly worse cardiac hemodynamics, including LA diameter, LV EF, pulmonary artery pressure, E/e′, LAA flow velocity, and SEC. Cardiac hemodynamic parameters were associated with increased risk of thromboembolic events (Table 1). However, the association between age and thromboembolic events was not fully explained by cardiac hemodynamics, since age was still an independent risk factor after adjusting echocardiographic and clinical parameters. In addition to age, E/e′ and LAA flow velocity were also independently associated with the risk of thromboembolic events. Our data suggest that worse cardiac hemodynamics in older patients partially explains the association between age and increased risk of thromboembolic events in patients with AF. However, additional underlying mechanisms remain unclear and must be elucidated in future studies.

## 5 LIMITATIONS

This study used the K‐NHIS sample cohort and KUMC registry data to perform retrospective analysis; therefore, is not free from the intrinsic limitations of such data. The K‐NHIS sample cohort data is based on an administrative database and is potentially susceptible to errors from inaccurate coding. Differentiation between paroxysmal and non‐paroxysmal AF was not possible in the K‐NHIS sample cohort data. Evaluation of cardiac imaging studies was also not possible. However, KUMC registry data included echocardiographic data. Furthermore, TEE was performed in a substantial number of patients, and important cardiac hemodynamic factors were evaluated. The K‐NHIS sample cohort data and KUMC registry do not represent the exact same population since the KUMC registry consists of patients with AF undergoing RFCA.

## 6 CONCLUSIONS

Age is a robust risk factor for thromboembolic events in patients with AF and older patients showed significantly worse cardiac hemodynamics. Despite equal CHA_2_DS_2_‐VASc score, the risk of thromboembolic events can be significantly different depending on age.

## CONFLICT OF INTEREST

The authors declare no potential conflict of interests.

## Supporting information


**Supplementary Table S1** Diagnosis codes.
**Supplementary Table S2.** Baseline characteristics of K‐NHIS sample cohort and KUMC registry.
