# Supplementary material for: Impact of age on thromboembolic events in patients with non‐valvular atrial fibrillation
Source: Clin Cardiol. 2019 Nov 15;43(1):78–85. doi: 10.1002/clc.23293 (PMC6954376; doi:10.1002/clc.23293)
Supplement: Supplementary file 1 — Supplementary Table S1 Diagnosis codes. Supplementary Table S2. Baseline characteristics of K‐NHIS sample cohort and KUMC registry. [file CLC-43-78-s001.docx]

**Impact of Age on Thromboembolic Events in Patients with Non-valvular Atrial Fibrillation**

Yun Gi Kim, MD^1^; Jong-Il Choi, MD, PhD, MHS*^1^; Ki Yung Boo, MD^1^; Do Young Kim, MD^1^; Yeji Hong, MSc^2^; Min Sun Kim, MSc^2^; Kwang-No Lee, MD^1^; Jaemin Shim, MD^1^; Jin Seok Kim, MD^1^; Young-Hoon Kim, MD^1^.

^1:^ Division of Cardiology, Department of Internal Medicine, Korea University Medicine Anam Hospital, Seoul, Republic of Korea

^2:^ Division of Medical Statistics, Korea University College of Medicine, Seoul, Republic of Korea

*** Address for correspondence**

Jong-Il Choi, MD, PhD, MHSc

Division of Cardiology, Department of Internal Medicine, Korea University College of Medicine and Korea University Medical Center, Seoul, Republic of Korea

73 Inchon-ro, Seongbuk-gu, Seoul 02841, Republic of Korea.

Tel: 82-2-920-5445

Fax: 82-2-927-1478

E-mail: jongilchoi@korea.ac.kr

**Running title:** Age and risk of thromboembolic events

**Supplementary Table S1.** Diagnosis codes.

| Disease | Diagnosis codes |
| --- | --- |
| Atrial fibrillation | I48.0, I48.1, I48.2, I48.3, I48.4, I48.9 |
| Exclusion criteria | Mitral stenosis (I05, I050, I052) and preexisting mechanical heart valves (Z952–Z954) |
| Hypertension | I10, I11, I12, I13, I15 |
| Diabetes mellitus | E10, E11, E12, E13, E14 |
| Heart failure | I42.0, I42.0A, I42.1, I42.1A, I42.2, I42.2A, I42.5, I42.6, I42.8, I42.9, I50.04, I50.08, I50.1, I50.9 |
| Ischemic stroke | I63 |
| Stroke, not specified as hemorrhage or infarction | I64 |
| Transient ischemic attack | G45.8, G45.9 |
| Arterial embolism | I74 |
| Vascular disease | I20, I21, I22, I24, I25 |

**Supplementary Table S2.** Baseline characteristics of K-NHIS sample cohort and KUMC registry.

|  | **K-NHIS sample cohort**  **(n = 5,896)** | | **KUMC registry**  **(n = 2,801)** |
| --- | --- | --- | --- |
| Age (years) | 63.17 ± 15.32* |  | 55.58 ± 10.97 |
|  | 0 – 19 | 62 (1.05%) |  |
|  | 20 – 24 | 57 (0.97%) |  |
|  | 25 – 29 | 67 (1.14%) |  |
|  | 30 – 34 | 103 (1.75%) |  |
|  | 35 – 39 | 173 (2.93%) |  |
|  | 40 – 44 | 242 (4.10%) |  |
|  | 45 – 49 | 382 (6.48%) |  |
|  | 50 – 54 | 561 (9.51%) |  |
|  | 55 – 59 | 570 (9.67%) |  |
|  | 60 – 64 | 666 (11.30%) |  |
|  | 65 – 69 | 810 (13.74%) |  |
|  | 70 – 74 | 834 (14.15%) |  |
|  | 75 – 79 | 655 (11.11%) |  |
|  | 80 – 84 | 397 (6.73%) |  |
|  | ≥ 85 | 317 (5.38%) |  |
| Male sex | 3,225 (54.70) |  | 2,217 (79.15) |
| Heart failure | 1,091 (18.50) |  | 139 (4.96) |
| Hypertension | 3,829 (64.94) |  | 1.054 (37.63) |
| Diabetes | 2,437 (41.33) |  | 266 (9.50) |
| Previous history of ischemic stroke, TIA, and systemic embolism | 470 (7.97) |  | 231 (8.25) |
| Vascular disease | 2,269 (38.48) |  | 226 (8.07) |
| Anticoagulation | 1,027 (17.42) |  |  |
| CHA_2_DS_2_-VASc | 2.99 ± 1.96 |  | 1.08 ± 1.10 |
| LA diameter |  |  | 41.16 ± 6.06 |
| LA volume |  |  | 92.98 ± 34.00 |
| LV ejection fraction |  |  | 54.77 ± 6.11 |
| E/e' |  |  | 8.79 **±** 3.38 |
| PAP |  |  | 30.63 ± 5.25 |
| LAA flow velocity |  |  | 48.55 ± 21.24 |

*Age in the K-NHIS sample cohort is categorized by 5-year intervals.

Results are presented as n (%) or mean with standard deviation.

AF: atrial fibrillation; BMI: body mass index; CVA: cerebrovascular accident; LA: left atrium; LAA: left atrial appendage; LR: late recurrence; LV: left ventricle; RFCA: radiofrequency catheter ablation; SEC: spontaneous echocontrast; TEE: transesophageal echocardiography; TIA: transient ischemic attack; TTE: transthoracic echocardiography; WBC: white blood cell.
